# Sublethal Dosage of Imidacloprid Reduces the Microglomerular Density of Honey Bee Mushroom Bodies

**DOI:** 10.1038/srep19298

**Published:** 2016-01-13

**Authors:** Yi-Chan Peng, En-Cheng Yang

**Affiliations:** 1 Department of Entomology, National Taiwan University, Taipei, Taiwan; 2 Graduate Institute of Brain and Mind Sciences, National Taiwan University, Taipei, Taiwan

## Abstract

The dramatic loss of honey bees is a major concern worldwide. Previous studies have indicated that neonicotinoid insecticides cause behavioural abnormalities and have proven that exposure to sublethal doses of imidacloprid during the larval stage decreases the olfactory learning ability of adults. The present study shows the effect of sublethal doses of imidacloprid on the neural development of the honey bee brain by immunolabelling synaptic units in the calyces of mushroom bodies. We found that the density of the synaptic units in the region of the calyces, which are responsible for olfactory and visual functions, decreased after being exposed to a sublethal dose of imidacloprid. This not only links a decrease in olfactory learning ability to abnormal neural connectivity but also provides evidence that imidacloprid damages the development of the nervous system in regions responsible for both olfaction and vision during the larval stage of the honey bee.

Severe loss of honey bees, commonly referred to as Colony Collapse Disorder (CCD), has occurred across Europe and North America^1^. Although this loss is believed to be the result of a combination of drivers^2^,^3^,^4^, chemical toxins such as imidacloprid, a new type of neonicotinoid pesticide, are thought to be a major contributor to the collapse of honey bee colonies. Neonicotinoid pesticides are a novel nicotinic analogue; a neurotoxin that binds to the nicotinic acetylcholine receptor (nAChR) of insects^5^,^6^, causing hyperexcitation that eventually leads to the insect’s death^7^.

Previous research has demonstrated the toxicity of imidacloprid to honey bees using common indicators of toxicity such as median lethal dose (LD_50_) and median lethal concentration (LC_50_)^8^,^9^,^10^,^11^,^12^. The oral LD_50_ of imidacloprid to honey bees is 3.7–40.9 ng/bee^9^. However, the sublethal dose of imidacloprid needs to be taken into consideration and tested as well, as honey bees in the field are not exposed to such high doses of pesticide. Studies have shown that the concentration of imidacloprid residue in soil, honey, and pollen is below 10 μg/kg^9^,^10^,^13^,^14^. Although this concentration is far less than the LC_50_, repetitive foraging on a low concentration of imidacloprid may result in an accumulation of the toxin. A sublethal dose of imidacloprid will severely affect a honey bee’s foraging behaviour by reducing the frequency of its foraging activity, resulting in foraging flights of extended duration^15^,^16^,^17^. In addition, a sublethal concentration of imidacloprid impairs the olfactory associative learning ability of honey bees^8^,^18^ and the fitness of honey bee colonies^19^.

In addition to honey bees coming into direct contact with imidacloprid as adults, they are also indirectly exposed to pesticides during their developmental stages, including their larval stage. Previous studies have demonstrated that a relatively high dose of imidacloprid residue in the hive delays the date of eclosion and reduces the longevity of the bees^20^,^21^, which affects the size and development of the colony^22^. By exposing a hive to imidacloprid, Yang *et al.*^23^ discovered that honey bee larvae fed with a sublethal dose of imidacloprid still turned into adult bees, but they did so with a decreased olfactory learning ability. This impairment occurred with a dose that could be as little as 0.04 ng per larva^23^. All of the above studies have demonstrated the sublethal effects of imidacloprid on bee colonies and on honey bee development.

This study aimed to identify the regions of the brain of adult bees that become impaired by the neurotoxin imidacloprid. The nAChRs are mainly located in the bilaterally symmetrical mushroom bodies (MBs)^24^,^25^, which are responsible for multisensory integration. MBs are thought to be related to learning, memory, cognitive processes, and the management of complex behaviours^26^,^27^,^28^,^29^,^30^, as well as the formation, consolidation, and recall of olfactory memories^31^,^32^,^33^. Among the functions in which MBs are involved, synaptic plasticity is a powerful mechanism that is the basis for coping with any task encountered and for making internal adjustments^34^,^35^.

Evaluation of the influence of a sublethal dose of imidacloprid during the larval stage on the adult bee brain^36^ requires analysis of the synaptic density^37^ or other neural structures^34^. The cup-shaped calyces in the MBs, which consist of many Kenyon cell dendrites, are important regions for sensory input^27^. Different areas in the calyces receive different types of sensory input: the lip regions mainly receive information from the olfactory system, the collar regions are stimulated by neurons from the optic ganglia, and the basal rings receive both visual and olfactory information^28^,^30^,^37^,^38^.

In the calyces, micro-glomerulus (MG), which consists of many synaptic units, have been chosen as the indicator of synaptic connectivity for distinct synaptic units^38^. They can be labelled by means of immunocytochemistry and are easily visualized and quantified^39^ (see Fig. 1). Many studies have used the number of MG as an indicator of neural connectivity during maturation^37^,^40^,^41^,^42^ and have shown a possible correlation between an increasing number of MG and age^42^. Accordingly, this study used the number of MG in the entire region of the calyces to identify whether a decrease in an adult bee’s olfactory learning ability correlates with the density of the synaptic units in the calyces.

## Results

### Standard trends in MG count, volumes of all subparts, and MG density in the calyces

Figures 2, 3, 4 show the standard trends in the MG count, volumes of all subparts, and MG density in the calyces of normal bees aged 1, 10, and 20 days (after eclosion) and in the foragers. Repeated-measures ANOVA was used to compare the differences in MG count, volumes of all subparts, and MG density among bees of different ages, except for the MG density in the collar (r, right) of RLC (right lateral calyx) and the lip (r) of RMC (right medial calyx), which were analyzed by GEE (generalized estimating equations). Regions that were bilaterally symmetrical showed similar trends in MG count, volumes of all subparts, and MG density.

The standard trends in MG count, volumes of all subparts, and MG density of the lateral calyces are shown in Figs 2, 3, 4. In all of the lips of the lateral calyces, the MG count and MG density increased significantly between the 1^st^ and 20^th^ days after eclosion (*p* < 0.05). A comparison of the MG count of the lateral calyces (except for the collar (l, left) of the RLC) showed significant growth between the 1^st^ and 20^th^ days after eclosion (*p* < 0.05). As for the volumes, only the basal ring, collar (r), and lip (r) of the LLC (left lateral calyx) showed no significant growth between the 1^st^ and 20^th^ day.

The standard trends in the MG count, volumes of all subparts, and MG density of the medial calyces are shown in Figs 2, 3, 4. All lips except for the lip (r) of LMC (left medial calyx) showed significant increases in MG density (*p* < 0.05).

The standard trends in the MG density of the calyces shown in Fig. 4 indicate that the 20^th^ day after eclosion is more suitable for representing the growth of the MG density than the 10^th^ day after eclosion. As the growth at the 20^th^ day tends to represent the highest density in all subparts, we chose samples from the 20^th^ day after eclosion to evaluate the effects of imidacloprid feeding.

### Decrease in MG density after feeding with a sublethal dose of imidacloprid

Figure 5 shows the effect of a sublethal dose of imidacloprid on the MG in the calyces 20 days after eclosion, using a confocal microscopy image to represent the decrease in MG density. The effect in the lip region is highlighted.

Tables 1-2 show a comparison of MG density in the calyces between the control group and the imidacloprid-fed groups using repeated-measures ANOVA, except for the basal ring of LLC, collar of LMC and lip (r) of RMC, which were analyzed using the Mann-Whitney *U* test. Each region was compared separately. When comparing normal bees with the imidacloprid-fed group, the MG density showed no significant differences among the regions in the calyces, indicating that the imidacloprid feeding process itself did not affect the MG density.

Table 1 shows a comparison of MG density in the lateral calyces between the control group and the imidacloprid-fed group. Almost all lips in the lateral calyces showed a significant decrease in MG density in bees treated with a 10 ppb dose of imidacloprid. However, the lip (r) in the LLC showed a significant decrease in MG density in bees treated with an imidacloprid concentration of 500 ppb. In the collars of the LLC, the MG density decreased significantly in bees that received a concentration of imidacloprid above 100 ppb. The collar (l) of the RLC showed a significant decrease in MG density in bees treated with an imidacloprid dose of 1 ppb. In the basal rings, the MG density of the LLC decreased significantly for bees treated with doses of imidacloprid of more than 100 ppb, while the MG density of the RLC showed a significant decrease in bees treated with doses of 10 ppb and higher.

Table 2 shows a comparison of MG density in the medial calyces between the control group and the groups fed with imidacloprid. In the LMC, the MG density in the lips decreased significantly in bees that received a dose of 500 ppb compared with the control group. The MG density in the collar decreased significantly for bees that received a dose of more than 100 ppb compared with the control group. In the RMC, the MG density in the lip (r) decreased significantly for bees that received a dose of more than 100 ppb compared with the control group, while the remaining subparts did not show a constant and significant decrease with increasing concentration. However, in the lip (l) of the RMC, the MG density decreased significantly for bees that received a dose of 1 ppb. The effects of different concentrations of imidacloprid on all regions of the four calyces are shown in Fig. 6.

## Discussion

### Standard trend in MG density in all calyces

This study is the first to observe a complete standard trend of the complete structure of the calyces of the adult bee. We established a standard trend for the MG count, volumes, and densities of all compartments in the calyces of bees from the 1^st^ day after eclosion to the foraging stage.

The lips, collars, and basal rings of the four calyces were studied using a confocal laser scanning microscope. The total number of images captured depended on the depth of the calyces and differed from brain to brain. Maleszka *et al.*^36^ indicated that the volume of MBs is not related to learning ability and that the degree of calyx depth varies among individual brains. Therefore, a better way to evaluate the neural connectivity is to use MG density as an indicator. This was also demonstrated by Groh *et al.*^37^.

With respect to the standard trend of MG density in the calyces, the results showed that not all MG density in the regions of the four calyces changed significantly with age (see Fig. 4). Among the lips, collars, and basal rings, all lips except lip (r) in the LMC showed a consistent trend of increasing MG density from the 1^st^ to 20^th^ day after eclosion, which is consistent with the study by Krofczik *et al.*^42^. As for the collars, the difference in MG density for the various ages was not obvious (see Fig. 4). The synaptic plasticity seemed to be more apparent in the lip regions, indicating that the ability to transfer olfactory information grows with age^38^. This result agrees with previous studies^43^,^44^,^45^, which demonstrated that olfactory learning ability increases with age.

In all collars except the right collar of the RLC, the MG density dropped significantly from the 1^st^ day to the 10^th^ day after eclosion (see Fig. 4). This decreasing trend was also observed in the basal rings of the lateral calyces (see Fig. 4). Surprisingly, during this time period, the MG density of the lip (r) in the LMC dropped significantly, and the lip (r) in the RMC showed a trend of decreasing MG density (see Fig. 4). MG density is expected to grow with age, as has been shown in a previous study^42^. Because MG density is calculated using the MG count and volume, a decrease in MG density could result from faster growth in volume compared to the MG count or from a decrease in the MG count, which may explain the decrease in MG density here.

In the collar (l) of the LLC and the collar (r) of the RLC, the volume grew faster than the MG count from the 1^st^ to the 10^th^ day after eclosion, causing a decrease in MG density (see Figs 2, 3, 4). In the collar (r) of the LLC, the collar (l) of the RLC, and the collars of the medial calyces, a drop in MG density was caused by a decrease in the MG count (see Figs 2, 3, 4). The reason for the decrease in the MG count during this period remains unknown. This decrease might result from the division of labour among honey bees. Generally, honey bees stay in the hive to serve as nurse bees between the 1^st^ and 11^th^ days after turning into an adult^46^. The bee hive is dark during this period, and the need to manage visual information is limited. The decrease in the MG density of the collars may be due to synaptic plasticity related to this environment^40^. In addition to honey bees^40^,^47^, ants^48^ also exhibit changes in the synaptic organization of mushroom bodies during different visual experiences and at different ages, showing that plasticity is a tool for adjusting to the environment.

### The effect of imidacloprid on MG density in the calyces

MG density decreased following treatment with different concentrations of imidacloprid in different regions. The effects differed at lower concentrations, but when the concentration reached 500 ppb, all regions were affected similarly (see Tables 1, 2, [Table t1], [Table t1], [Table t1], [Table t2]). Figure 5 shows which regions in all four calyces were affected following treatment with different concentrations of imidacloprid.

Previous studies have shown that the concentration of imidacloprid residue in soil, honey, and pollen is below 10 ppb^9^,^13^,^14^. This concentration has already been shown to affect adult bees’ olfactory learning ability following exposure in the larval stage^23^. In the present study, we found that a dose of 10 ppb caused the MG density in the calyces of the adult bees to decrease significantly. The calyces are important sensory input regions in the mushroom bodies. It is therefore reasonable to speculate that exposure to 10 ppb of imidacloprid during the larval stage affects the MG density in the calyces of the MBs and impairs the olfactory learning ability of the adult bee. However, we cannot exclude the possibility that the olfactory pathway between the olfactory receptors and the antenna lobes was impaired.

In addition to the olfactory aspect, the present study also demonstrates that the MG density in the collar, which is where visual information is received^38^, decreased in bees treated with a sublethal dose of imidacloprid. It is worth noting that the impairment of olfactory and visual input was already apparent at a dose of 1 ppb of imidacloprid in three subparts. In addition, the basal ring, which receives both olfactory and visual information, was affected (see Table 1). With both the olfactory and visual input regions in the calyces affected by this neurotoxin, it can be assumed that the behaviour of these honey bees would have been substantially affected in a negative way.

### Foraging activity is dependent on synaptic connectivity

Previous studies have demonstrated that foraging behaviour is highly correlated with changes in plastic structures in the MBs^34^,^35^,^37^,^42^. Previous studies have also shown that age and behavioural experience affect the number of MG in the MBs^40^,^42^,^47^, indicating that plasticity in the MBs is needed during honey bee maturation to attain an optimal condition. The abnormal neural connectivity that results from contamination by imidacloprid during the larval stage affects not only behaviour but also the neural development of the brain. In addition, a previous study indicated that MG density in the MBs increases when a specific olfactory long-term memory (LTM) is formed, and this memory consolidation requires gene transcription^41^. During LTM consolidation, the synaptic rearrangement may include the formation of new synapses in the MBs^49^ as well as a stable increase in MG density^41^. This clearly indicates that exposure to imidacloprid causes the MG density to decrease, and this might also affect the formation of LTM.

LTM is important for honey bees during foraging activity. When foraging, foragers are able to efficiently learn cues from different flowers because of the reward they provide^50^. Foraging activity is guided by the benefits the foragers obtain through specific foraging behaviour^51^. Flowers appear in patches distributed at various distances, which can be categorized by the various travel times between them^50^. These time intervals may vary from minutes to months. In addition, flower fidelity is guided by the bees’ LTM^50^. When their LTM is damaged by imidacloprid, honey bees are likely to become lost while foraging.

### Impairment of neural connectivity

To conclude, the decrease in MG density in the calyces of a honey bee after having been exposed to imidacloprid during the larval stage might affect the adult in various ways, leading to diminished olfactory learning ability, reduced LTM, and consequently limited foraging efficiency. From both a behavioural and a neurophysiological perspective, the evidence supports the conclusion that a sublethal dose of imidacloprid negatively affects the development of the honey bee nervous system. It is thus reasonable to speculate that imidacloprid residue in the environement would impair bees’ neural connectivity, eventually resulting in the decline and collapse of colonies. The decrease in MG density in the calyces of honey bees has a profound impact on their nervous system and behaviour.

## Materials and Methods

### Animals

This research was conducted using honey bees (*Apis mellifera* L.) reared at National Taiwan University. Each bee colony tested was evaluated as stable, and consisted of a normal egg-laying queen, larvae, pupae, honey, and pollen. All colonies were monitored regularly to ensure that they were in optimum condition. If required, a 50% sucrose solution and artificial pollen were fed to a colony to maintain its status.

In our experiment, the honey bees were divided into two groups: the normal honey bees and the honey bees that were fed a sublethal dose of imidacloprid during their larval stage.

The normal honey bees were collected by choosing one comb with a large number of capped-brood cells. This comb was then placed in an incubator, and the temperature was maintained at 33–34 °C. After the honey bees emerged, they were immediately marked with acrylic paint on their thoraxes for identification of their eclosion date and were then put back into their hive. Twenty honey bees of each age (1 day, 10 days, and 20 days) were randomly collected. In addition, 20 foragers carrying pollen on their corbiculate legs were collected outside the hive. The collecting method for honey bees fed with imidacloprid is described below.

### Preparation of the imidacloprid solution

Imidacloprid (95% TG, Bayer Cropscience AG, Monheim am Rhein, Germany) is a powder and was dissolved in dimethyl sulfoxide (DMSO, MP Biomedical). To reduce the effect of solvent on honey bees, the solvent used should not induce any abnormal behaviours in the honey bees. Previous studies have indicated that acetone significantly reduces the feeding activity of honey bees^17^, whereas DMSO does not. For this reason, DMSO was chosen as the solvent for imidacloprid. The amount of solvent remaining in the imidacloprid solution was trivial (below 0.0005%, v/v, which has been shown to be negligible in previous studies^8^,^11^,^12^,^14^,^15^,^18^).

Yang *et al.*^23^ demonstrated that a dose of imidacloprid above 0.04 ng would result in an apparent reduction in the bees’ olfactory associative ability after they matured into adults. Therefore, bees that received 0.04 or 0.4 ng of imidacloprid were deemed to have impaired olfactory learning ability and were designated as the experimental group. Bees receiving 0.004 ng of imidacloprid were designated as the group where the olfactory learning ability of the adult bee was not impaired. This was done to compare the density of synaptic units in the calyces of the mushroom bodies among the various groups. Yang *et al.*^23^ showed that capped-brood rate, pupation rate, and eclosion rate were not affected by a dose of up to 200 ng. Therefore, in the experimental group, the comparatively higher dose of 2 ng imidacloprid, which did not affect the survival rate of the larvae, was added to test whether the development of neural connectivity in the brains of the worker bees was normal.

Thus, the concentrations of imidacloprid used in our experiment were: 1 μg/L (ppb), 10 μg/L, 100 μg/L, and 500 μg/L, respectively. All concentrations used were diluted from the imidacloprid stock solution, which was prepared using a mixture of 0.5 mL distilled deionized water (DDW) and 0.5 mL DMSO to which 1 mg of imidacloprid powder was added, giving a concentration of 1 g/L. Then, 2 μL of the stock solution was added to 1998 μL of DDW, resulting in an intermediate concentration of 1000 μg/L. The test solutions in concentrations of 500 μg/L, 100 μg/L, 10 μg/L, and 1 μg/L of imidacloprid were obtained by using 1000 μL, 200 μL, 20 μL, and 2 μL of intermediate-concentration solution added to 1000 μL, 1800 μL, 1980 μL, and 1998 μL of DDW, respectively.

### Imidacloprid feeding process

After the appropriate colonies were selected, the queen of each colony was restricted to laying her eggs on one honeycomb within her hive so that the offspring’s age could easily be determined after they turned into larvae. The areas of the cells used for testing 1-day-old larvae were labelled by marking transparent slides placed on the honeycomb. The larvae were divided into five groups, one for the control group and the others for the experimental groups. It is typical for a normal egg-hatching queen to produce approximately 400 one-day-old larvae. These larvae were located on one side of the comb and were properly marked^23^.

After the marking procedure, the experimental groups were fed 1 μL of 1 μg/L, 10 μg/L, 100 μg/L, and 500 μg/L concentration of imidacloprid, respectively, while the control group was fed a 0.0005% DMSO solution (the actual concentrations of DMSO fed to the larvae were 0.000001%, 0.00001%, 0.0001%, and 0.0005%, respectively). As mentioned earlier, the negligible concentration of DMSO can be ignored, and therefore the highest concentration of DMSO in the experimental group (0.0005%) was chosen to be fed to the control group. This solution was supplied to the larvae near the cell wall by means of a pipette. This feeding procedure was repeated daily for four days, resulting in a total intake of imidacloprid in the experimental groups of 0.004, 0.04, 0.4 and 2 ng/larva, respectively. We excluded those larvae that did not complete the four-day feeding process, either due to prior capping or death.

Usually, the cells are capped after six days, and the larvae turn to pupae. To avoid any prior disturbance of the pupae and to ensure their stable state, we waited approximately seven days before transferring them into a 96-well plate and placing it in an incubator at 34 °C and a relative humidity of 70–80%. The pupae were placed in different 96-well plates that were separated in different compartments according to the concentration of imidacloprid with which they were fed, to avoid any mix-up after they reached the adult stage. Approximately six days after capping, the pupae became adults and emerged from their cells. After eclosion, the adult bees were marked on the thorax with acrylic paint of a different colour, depending on the treatment group to which they belonged, and were then returned to their original hive. After excluding the dead, the average number of marked bees in each group was approximately fifty. Twenty adult bees from each group were then randomly collected on the 20^th^ day after eclosion. These bees underwent an antibody staining procedure to reveal the density of their MG after being fed a sublethal dose of imidacloprid during their larval stage.

### Immunocytochemistry

To analyse the effect of imidacloprid during the larval stage of honey bees on the MG in their calyces, we followed the protocol of Groh *et al.*^47^, but with some adjustments to be able to immunolabel the synaptic units in a whole-mount preparation. The tested bees were anaesthetized at 4 °C, and their heads were fixed using beeswax. After removing the setae on the head capsule, the head was cut open along the edge, and the glandular tissue, tracheae, and membrane were exposed. The head was covered with cold physiological saline (130 mM NaCl, 5 mM KCl, 4 mM MgCl_2_, 5 mM CaCl_2_, 15 mM HEPES, 25 mM glucose, 160 mM sucrose; pH 7.2)^47^ and the glandular tissue, tracheae, and membrane were removed, as well as the ocelli.

The head was then cut from the honey bee’s body and pre-fixed in 4% paraformaldehyde (Riedel-de Haën, 16005) at 4 °C in 0.01 M phosphate-buffered saline (PBS; 0.01 M phosphate buffer, 0.0027 M potassium chloride and 0.137 M sodium chloride, pH 7.2) for 30 minutes^52^. The fixed brain was then dissected out from the head capsules under PBS. After a quick rinse in PBS, the brain was then placed in an Eppendorf tube and fixed in PFA for 20 hours at room temperature^53^.

The brains were washed in PBS five times (each time for 10 minutes) and were then permeabilized in 0.2% Triton X-100 (Tx) in PBS twice for 10 minutes each. They were then blocked in 2% normal goat serum (NGS; 005-000-121, Jackson ImmunoResearch Laboratories) in 0.2% PBS-Tx for 3 hours at 23 °C^47^.

For the synapse labelling process, the brains were incubated in the primary antibody SYNORF1 (monoclonal antibody against the *Drosophila* synaptic-vesicle-associated protein synapsin I (Erich Buchner, Wüerzburg, Germany)^52^,^54^), which was diluted 1:10 in 0.2% PBS-Tx with 2% NGS for four nights at 4 °C^47^. This antibody is commonly used in the synaptic immunostaining of *Drosophila* and honey bees^37^,^40^.

After being rinsed in PBS five times, for 10 minutes each time, the brains were incubated in goat anti-mouse DyLight 550 secondary antibody (Mouse IgG-heavy and light chain cross-adsorbed Antibody; Catalogue No. A90-516D3, Bethyl Laboratories, Inc.; diluted with 1:250 in PBS) for four nights at 4 °C^47^. Afterwards, they were washed in PBS five times, for 10 minutes each time, and dehydrated in an ascending ethanol series (30%, 50%, 70%, 90%, 95%, 3X 100%). Finally, the brains were cleared in methyl-salicylate (M6752, Sigma-Aldrich, Germany) and mounted whole-mount in Permount (SP15-100; Fisher Scientific, Fair Lawn, NJ) on a modified concave slide (Marienfeld, Lauda-Königshofen, Germany). Finally, they were covered with cover glasses (thickness No. 1)^52^.

### Confocal microscopy

The whole-mount preparations were scanned using a Laser Scanning Confocal Microscope (Leica TCS SP5 II, Germany) with a 20X objective lens (HCX PL APO CS 20X/0.70 DRY, WD = 0.59 mm), a 40X objective lens (HCX PL APO CS 40X/1.25 OIL, WD = 0.1 mm, UV/405 nm) and a 63X objective lens (HCX PL APO lambda blue 63X/1.40 OIL, WD = 0.1 mm, UV/405 nm). Synaptic units labelled with goat anti-mouse DyLight 550 secondary antibody were excited at 543 nm using a HeNe laser and emitted in the range of 560–580 nm. The scanning was performed at a resolution of 1024 × 1024 pixels at an acquisition speed of 100 Hz.

**Figure 1:**
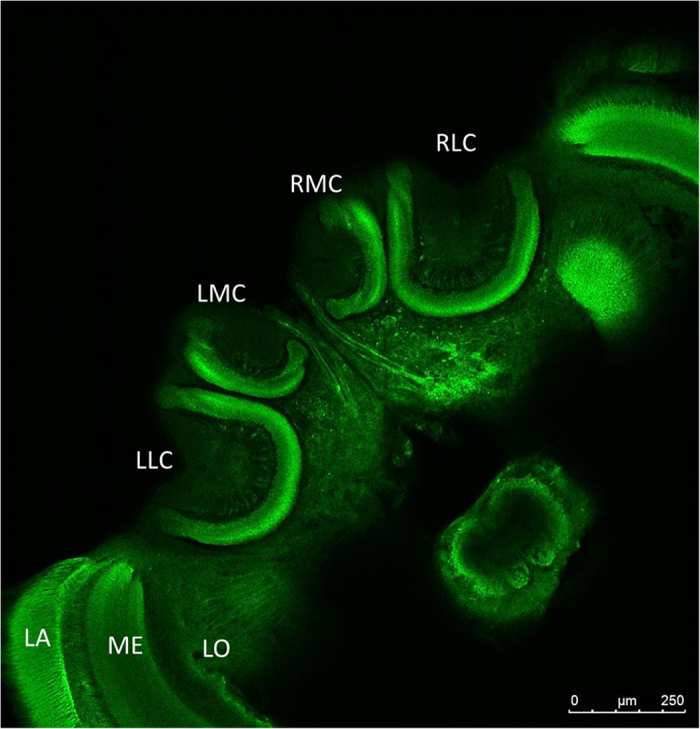
A confocal section of the frontal view of the centre of an adult bee’s brain immunolabelled for synaptic units. Immunolabelling of the MG (green) in the calyces of the mushroom bodies of the honey bee’s brain: LA, lamina; LLC, left lateral calyx; LMC, left medial calyx; LO, lobula; ME, medulla; RMC, right medial calyx; RLC, right lateral calyx.

**Figure 2:**
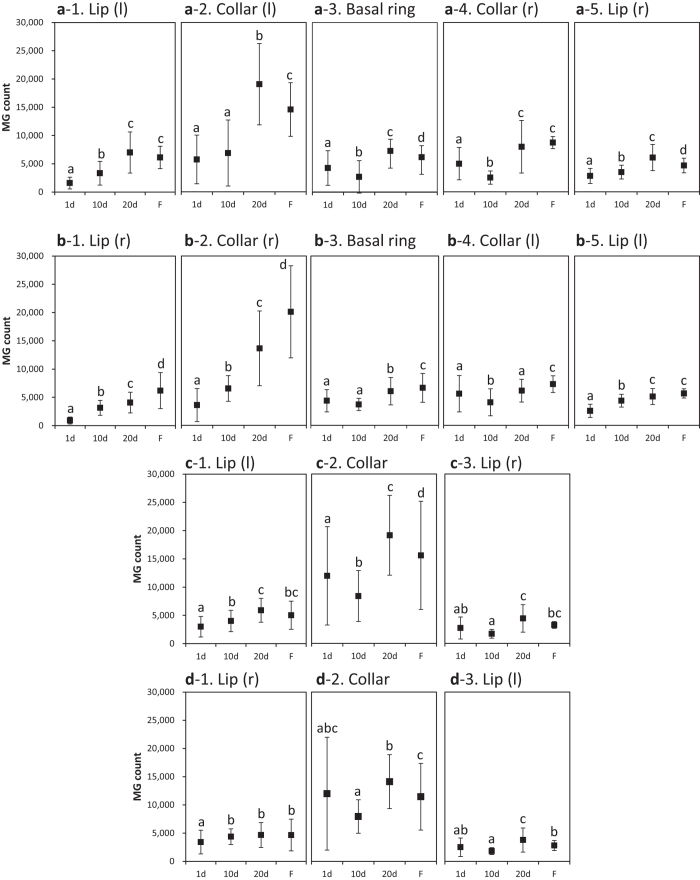
The standard trend in the MG count for bees aged 1 to 20 days (after eclosion) and for foragers in each region of the four calyces (LLC: **a**1–5; RLC: **b**1–5; LMC: **c**1–3; RMC: **d**1–3). Regions that are bilaterally symmetrical are shown in the same column. Different letters show significant differences. Error bars are SD. d, days after eclosion; F, foragers; l, left; MG, micro-glomerulus; r, right.

**Figure 3:**
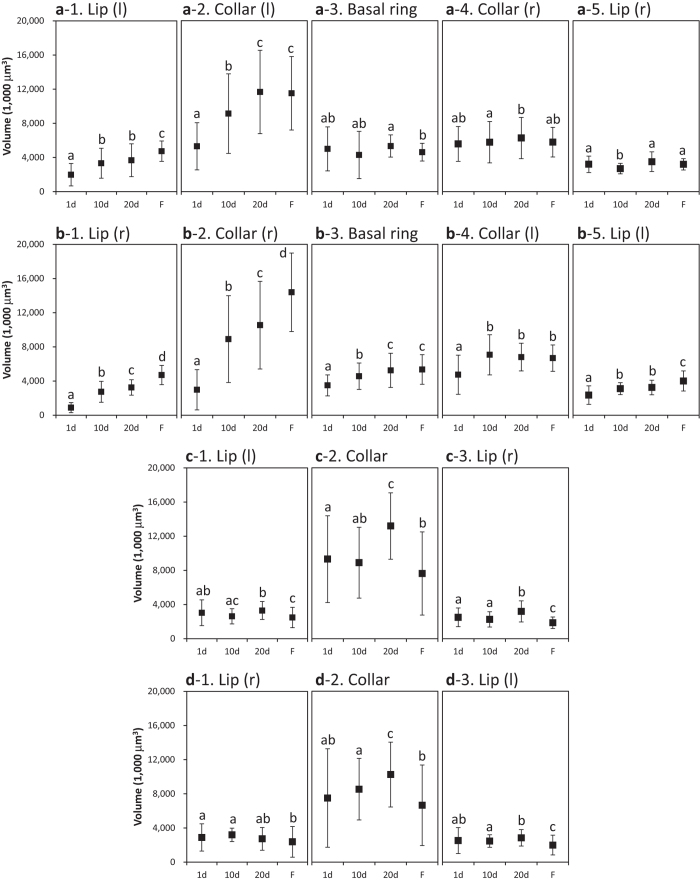
The standard trend in the volumes of different compartments for bees aged 1 to 20 days (after eclosion) and for foragers in each region of the four calyces (LLC: **a**1–5; RLC: **b**1–5; LMC: **c**1–3; RMC: **d**1–3). Regions that are bilaterally symmetrical are shown in the same column. Different letters show significant differences. Error bars are SD. d, days after eclosion; F, foragers; l, left; MG, micro-glomerulus; r, right.

**Figure 4:**
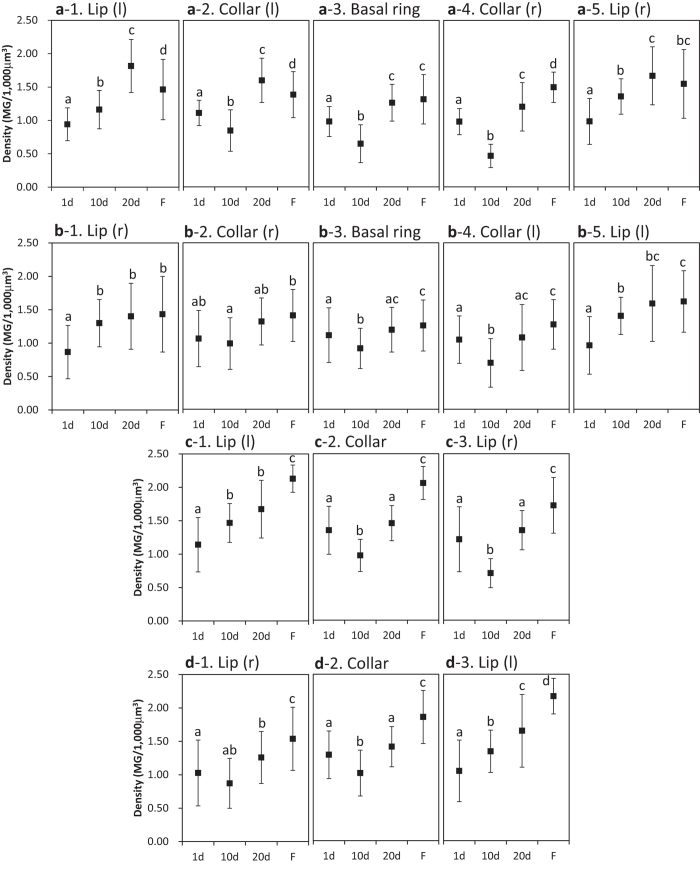
Standard trends in the MG density for bees aged 1 to 20 days (after eclosion) and for foragers in each region of the four calyces (LLC: **a**1–5; RLC: **b**1–5; LMC: **c**1–3; RMC: **d**1–3). Regions that are bilaterally symmetrical are shown in the same column. Different letters show significant differences. Error bars are SD. d, days after eclosion; F, foragers; l, left; MG, micro-glomerulus; r, right.

The calyces are located on the upper surface of the brain. This methodology allowed the scans to go through the entire structure of the calyces from top to bottom. Scans were taken at 5 μm intervals to measure the MG in the calyx region^47^. Each brain contains four calyces (Fig. 1). In the scanning process, the structure of each of the four calyces was scanned separately to obtain a scan of the complete structure. The total number of images captured for each calyx differed from brain to brain and depended on the depth of the calyx.

### Image processing

The confocal images were processed by Imaris^®^ (x64 version 7.6.5, Bitplane, Zürich, Switzerland). The images for the entire structure of the calyces were processed to obtain a 3D structure of each calyx formed by the many layers of images taken by confocal microscopy. By using this software, the 3D structural diagram could be framed and separated into different regions of interest by creating a new surface covering that specific part, such as the lips, collars, and basal rings. The software then allowed us to view the 3D structural diagram of the original layers taken by the confocal microscope. Using these images, we started at the layer from which the boundaries of all the subparts in the calyces could be easily identified. The lip regions were the apparent ellipse on the two starting points of the U-shape. The boundary of the total collar region was determined by the line of the dense part in the collar because of its obvious borderlines. The rest was shown as the basal ring. Framing these subparts layer by layer, we reconstructed the 3D structural diagram of the calyces with different subparts shown with different surfaces. Using this software, we defined the lateral calyces with two lips, two collars, and a basal ring (from left (l) to right (r): lip (l), collar (l), basal ring, collar (r), and lip (r)) and the medial calyces with two lips and a collar (lip (l), collar, and lip (r)). Different treatments were randomly assigned as numbers without indicating the treatment group to which they belonged.

### Data acquisition of MG counts

The MG counts for all the subparts of the calyces were determined by the Imaris^®^ surface function. The surface function is created by framing each specific region layer by layer through the 3D structure. The diameter range of the MG was defined as being between 1.5 and 3.5 μm. This range was set by measurement of the MG through the Imaris^®^ function. In addition, setting the diameter lower than 1.5 μm introduced background noise, while setting it to a value greater than 3.5 μm could cause miscalculation by overlapping parts that are not relevant for counting MG. We therefore chose this range of diameters to define the area for the MG count. The results—which processed the MG depicted as dots—were visually confirmed to ensure that all defined MGs in this diameter range were counted within the selected threshold range. Although previous studies have indicated that the human eye is the best tool for synapse identification because of the perception and expertise of the human viewer in defining the presence of labels^55^, the large amount of data in this study could not be processed entirely by the human eye.

We utilized the Imaris^®^ software to calculate the enormous number of MG within each calyx using the filters to set the threshold of the MG-counting area. Through surface function framing of each specific subpart, the volume was calculated by analysing the entire surface region. The MG density was calculated by using the MG count and the calculated volume of each region.

### Statistical analysis

Ten samples each were analysed from the group of normal bees and the experimental groups. To avoid bias caused by individual differences, the highest and lowest density values in each group were excluded from the statistical analysis.

The standard trends in all regions of the calyces for the group of normal bees were set by bees aged 1, 10, and 20 days after eclosion and foragers. The statistical values of spots, volumes, and density were square-root transformed before analysis to conform to the assumption of a normal distribution when using Kolmogorov-Smirnov tests. To compare the differences in MG count, volumes of the different compartments, and the density among bees of different ages, data were analysed using repeated-measures ANOVA when values fit normal distributions or GEE tests when they did not fit normal distributions. The comparisons between the control group and each imidacloprid-fed group in all regions were statistically analysed using the repeated-measures ANOVA when values fit normal distributions or the Mann-Whitney *U* test when they did not fit normal distributions.

**Figure 5:**
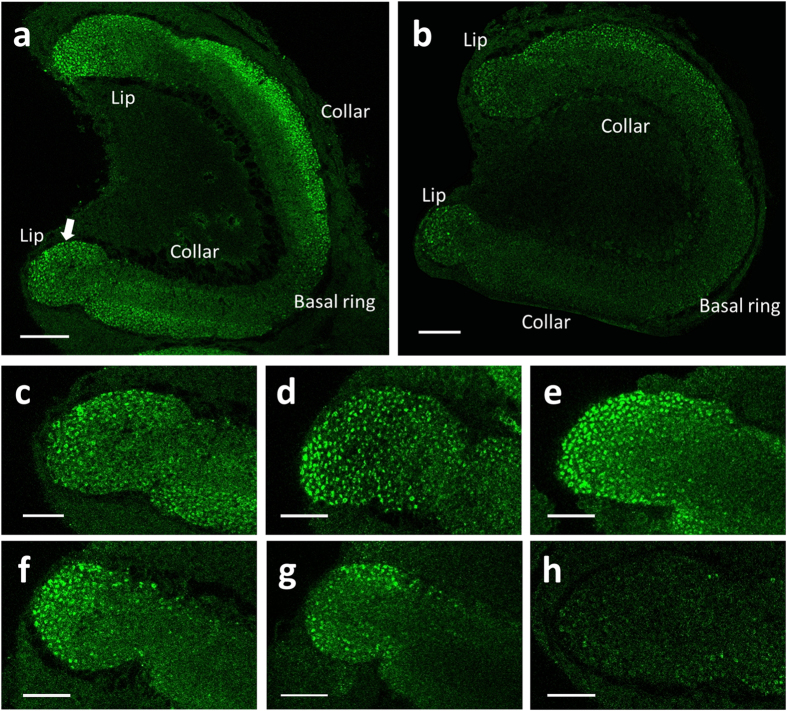
Effect of imidacloprid feeding on the calyces during the larval stage at the 20^th^ day after eclosion in the mushroom bodies of an adult bee. This figure shows the immunolabelling of micro-glomerulus in the RLC. The arrow in **a** shows the MG (immunolabelled as green). (**a** and **b)** The effect of imidacloprid in the RLC. (**a)** Normal adult bee; (**b**) adult bee fed 500 ppb of imidacloprid during the larval stage. (**c–h**) The effect of imidacloprid in the lip (l) of the RLC. Figures (**c–h)** are normal bees, control, adult bee fed 1 ppb, 10 ppb, 100 ppb, and 500 ppb of imidacloprid, respectively, during the larval stage. The scale bar = 50 μm in (**a)** and (**b)** 25 μm in (**c–h)**.

**Figure 6:**
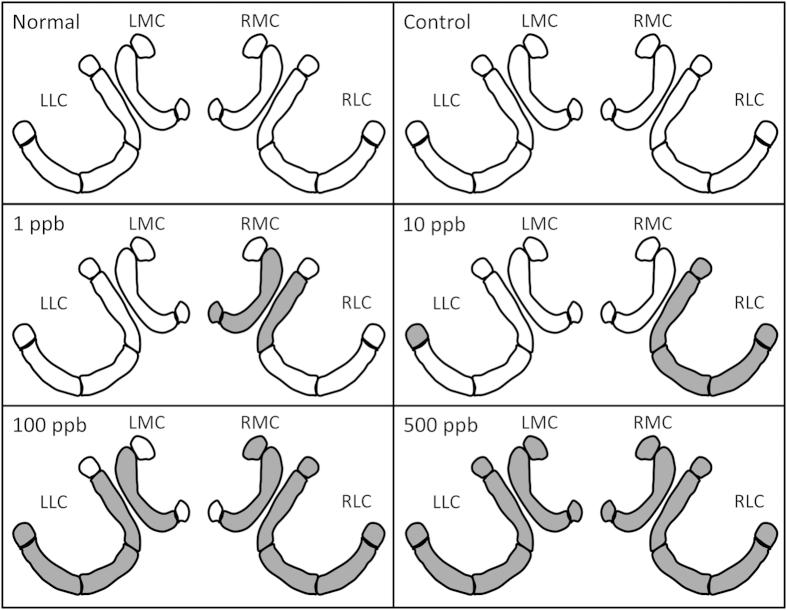
The effects of different concentrations of imidacloprid on all regions of the four calyces. The grey regions show where there are significant differences between the control group and the groups with specific concentrations.

Statistical analyses were performed with SPSS 21.0 software. For ease of interpretation, all figures and tables present raw data as the means ± standard deviation (SD). The brightness and contrast of the images presented in Figs 1 and 5 were slightly adjusted for better indication.

## Additional Information

**How to cite this article**: Peng, Y.-C. and Yang, E.-C. Sublethal Dosage of Imidacloprid Reduces the Microglomerular Density of Honey Bee Mushroom Bodies. *Sci. Rep.* **6**, 19298; doi: 10.1038/srep19298 (2016).

## Figures and Tables

**Table 1 t1:** A comparison of MG density in the LLC and RLC between the control group and the groups fed imidacloprid.

Density^1,2,3^					
Treatment (conc.^4^)	LLC				
	Lip (l)^4^	Collar (l)	Basal ring	Collar (r)^4^	Lip (r)
Normal	1.82 ± 0.40	1.60 ± 0.33	1.26 ± 0.28	1.20 ± 0.36	1.67 ± 0.44
Control	2.01 ± 0.26	1.48 ± 0.45	1.39 ± 0.25	1.18 ± 0.37	1.97 ± 0.24
1 ppb	1.82 ± 0.61	1.17 ± 0.58	1.14 ± 0.56	0.90 ± 0.53	1.91 ± 0.45
10 ppb	1.46 ± 0.31*	1.12 ± 0.28	1.18 ± 0.46	1.06 ± 0.34	1.85 ± 0.32
100 ppb	1.35 ± 0.49**	0.84 ± 0.36**	0.84 ± 0.26**	0.68 ± 0.18**	1.72 ± 0.31
500 ppb	0.93 ± 0.26***	0.78 ± 0.40**	0.58 ± 0.45**	0.38 ± 0.25***	0.81 ± 0.35***
Treatment (conc.^4^)	RLC				
	Lip (r)^4^	Collar (r)	Basal ring	Collar (l)^4^	Lip (l)
Normal	1.40 ± 0.49	1.32 ± 0.35	1.19 ± 0.34	1.08 ± 0.49	1.59 ± 0.57
Control	1.66 ± 0.41	1.29 ± 0.36	1.28 ± 0.27	1.01 ± 0.30	1.93 ± 0.27
1 ppb	1.36 ± 0.37	1.07 ± 0.35	0.99 ± 0.47	0.57 ± 0.28**	1.72 ± 0.38
10 ppb	1.13 ± 0.36*	0.82 ± 0.26*	0.81 ± 0.28*	0.64 ± 0.15*	1.52 ± 0.21*
100 ppb	0.90 ± 0.58**	0.53 ± 0.35**	0.62 ± 0.39**	0.48 ± 0.25**	1.49 ± 0.39*
500 ppb	0.78 ± 0.60**	0.77 ± 0.70*	0.63 ± 0.58**	0.29 ± 0.28***	0.85 ± 0.45***

^1^Data are expressed as the mean ± SD. Bees were collected on the 20^th^ day after eclosion. The regions in the LLC and RLC that are bilaterally symmetrical are shown in the same column.

^2^MG (micro-glomerulus)/1000 μm^3^.

^3^*p < 0.05, **p < 0.01, ***p < 0.001.

^4^conc., concentration; l, left; r, right.

**Table 2 t2:** A comparison of the MG density in the LMC and RMC between the control group and the groups fed imidacloprid.

Density^1,2,3^			
Treatment (conc.^4^)	LMC		
	Lip (l)^4^	Collar	Lip (r)^4^
Normal	1.67 ± 0.43	1.46 ± 0.26	1.35 ± 0.29
Control	1.91 ± 0.42	1.47 ± 0.38	1.40 ± 0.39
1 ppb	1.84 ± 0.44	1.23 ± 0.53	1.14 ± 0.40
10 ppb	1.66 ± 0.49	1.35 ± 0.50	1.16 ± 0.42
100 ppb	1.64 ± 0.55	1.04 ± 0.35*	1.34 ± 0.46
500 ppb	1.40 ± 0.37*	0.75 ± 0.42**	0.95 ± 0.41*
Treatment (conc.^4^)	RMC		
	Lip (r)*	Collar	Lip (l)*
Normal	1.65 ± 0.55	1.41 ± 0.30	1.26 ± 0.39
Control	1.93 ± 0.37	1.43 ± 0.46	1.61 ± 0.31
1 ppb	1.90 ± 0.44	1.01 ± 0.46*	1.06 ± 0.45*
10 ppb	1.76 ± 0.35	1.13 ± 0.24	1.25 ± 0.43
100 ppb	1.43 ± 0.56*	0.86 ± 0.37**	1.32 ± 0.44
500 ppb	1.29 ± 0.47*	0.86 ± 0.56**	1.02 ± 0.47**

^1^Data are expressed as the mean ± SD. Bees were collected on the 20^th^ day after eclosion. The regions in the LMC and RMC that are bilaterally symmetrical are shown in the same column.

^2^MG (micro-glomerulus)/1000 μm^3^.

^3^*p < 0.05, **p < 0.01, ***p < 0.001.

^4^conc., concentration; l, left; r, right.
